# Identification of 17q12 microdeletion syndrome in a Latin American patient with maturity-onset diabetes of the young subtype 5: a case report

**DOI:** 10.1186/s13256-023-03873-6

**Published:** 2023-04-05

**Authors:** Guillermo Edinson Guzmán, Ithzayana Madariaga, Carlos Julio Vargas, Laura Ballen Galeano, Maria Angélica Guerra, Jose Antonio Nastasi

**Affiliations:** 1 Departamento de Endocrinología, Fundación Valle del Lili, Calle 18 No. 122-135, 760032 Cali, Colombia; 2 Facultad de Ciencias de la Salud, Universidad Icesi, Calle 18 No. 122-135, 760032 Cali, Colombia; 3 Centro de Investigaciones Clínicas, Fundación Valle del Lili, Cra 98 No.18-49, 760032 Cali, Colombia; 4 Departamento de Genética, Fundación Valle del Lili, Calle 18 No. 122-135, 760032 Cali, Colombia

**Keywords:** MODY, Microdeletion syndrome, Monogenic disease, Latin American population, Colombian population, Case report

## Abstract

**Background:**

Maturity-onset diabetes of the young comprises a large group of autosomal inherited gene mutations. Maturity-onset diabetes of the young subtype 5 is caused by mutations in the *HNF1B* gene. This gene is expressed in the early phase of embryonic development in the pancreas, kidneys, liver, and genital tract; therefore, kidney or urinary tract malformations are associated with diabetes mellitus. The 17q12 deletion syndrome is a cause of maturity-onset diabetes of the young subtype 5 that should be considered.

**Case presentation:**

We present the case of a 35-year-old Hispanic female patient with a history of bicornuate uterus and polycystic renal disease that required kidney transplant. She had insulin-dependent diabetes, with her mother, maternal grandmother, and great-grandmother showing a similar clinical manifestation. Molecular analysis showed a deletion in chromosome 17q12 involving 15 genes, including *HNF1B*. Therefore, a diagnosis of deletion syndrome was made.

**Conclusions:**

The 17q12 deletion syndrome represents a rare genetic syndrome that involves different genes, including *HNF1B*. Principally, it is characterized by the combination of genitourinary tract malformations and diabetes mellitus, similar to our patient.

## Highlights

- MODY subtype 5 is caused by mutations in the *HNF1B* gene.
- MODY should be suspected in individuals who are lean and lack ethnic T2DM risk factors.
- Deletion syndrome has different phenotypic expressions based on the genes involved.

## Introduction

Diabetes mellitus (DM) has a worldwide distribution and an increasing incidence. The most prevalent types are type 1 (T1DM) and type 2 (T2DM). Each type has a precise etiology (autoimmune mechanisms and multifactorial origin, respectively) and clinical characteristics [1, 2]. There is a third category of diabetes with specific etiologies, including diabetes secondary to a drug, transplant, injury, or other genetic or nongenetic disease. Maturity-onset diabetes of the young (MODY) is one of the most well-known forms of monogenic diabetes.

MODY classically presents in individuals with hyperglycemia before the age of 25 years, does not require insulin, and has evidence of autosomal dominant inheritance [3]. MODY is particularly suspected in individuals who are lean and not from ethnic groups with a high prevalence of type 2 diabetes (for example, African–American, Hispanic, Pacific Islander). Lack of these T2DM risk factors and T1DM-specific markers, including diabetes autoantibodies and low C-peptide levels (as a measure of endogenous insulin production), indicates a high probability of MODY. Nonetheless, genetic testing is necessary to diagnose MODY [2].

MODY involves a wide group of genetic mutations with autosomal dominant inheritance [2]. MODY subtype 5 is caused by deletions in the *HNF1B* gene, located on chromosome 17q12. This gene is expressed in the early phase of embryonic development in the pancreas, kidneys, liver, and genital tract. Its prevalence is low within the already small prevalence of MODY [4]. *HNF1B* deletion has recently been shown to be associated with 17q12 deletion syndrome in virtually all cases [5]. We discuss a rare case of microdeletion syndrome in a Latin American patient.

## Case presentation

**Fig. 1:**
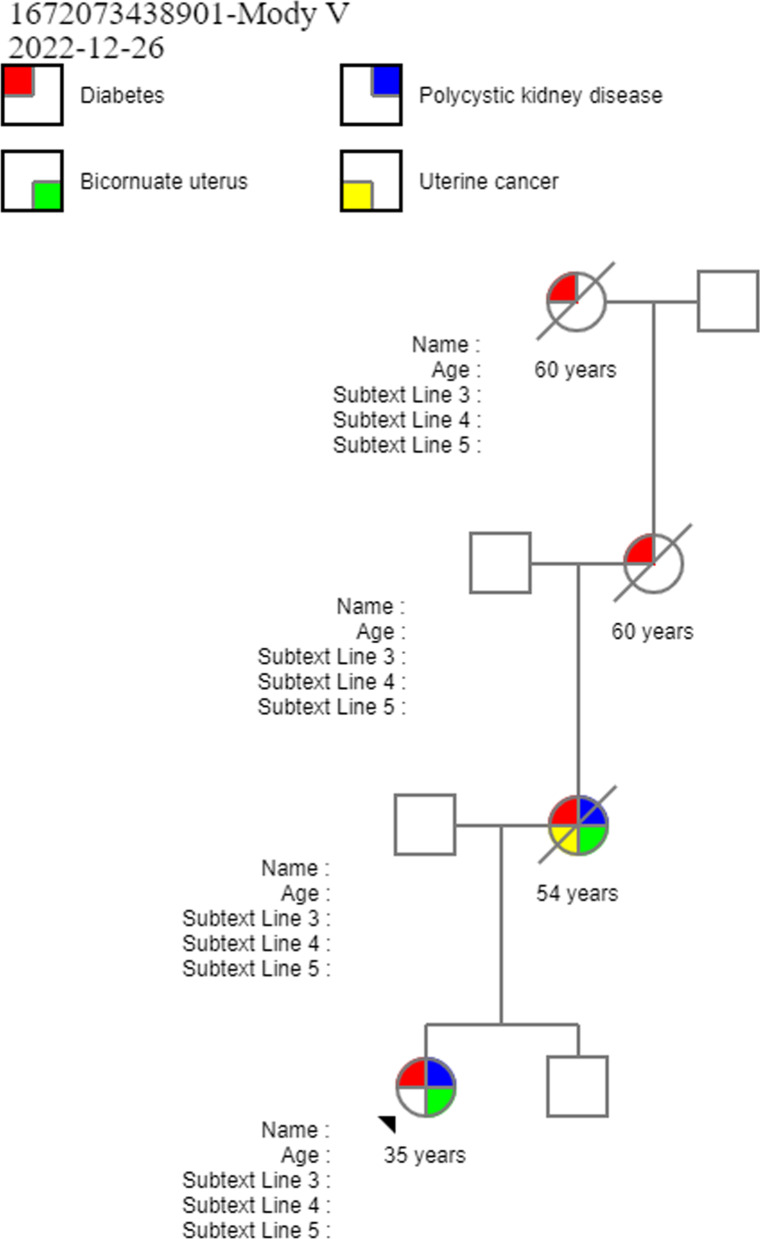
The patient’s family tree. Mother diagnosed with diabetes, polycystic kidney, and bicornuate uterus; grandmother and great-grandmother with a history of early onset diabetes

A 35-year-old Hispanic woman with mild mental retardation was referred for poorly controlled DM. She was diagnosed with polycystic kidney disease and DM at ages 9 and 11 years, respectively. She subsequently developed a rapid progression to diabetic nephropathy, requiring a living donor kidney transplant at age 12. In her early 30s, she was diagnosed with a bicornuate uterus. Within her family history, her mother, maternal grandmother, and great-grandmother also had early onset diabetes with death before 60 years of age (Fig. 1). No parental consanguinity was noted.

On physical examination, the patient had a blood pressure of 140/80 mmHg, a body mass index (BMI) of 30.1 kg/m^2^, absence of eyebrow roots, widow’s peak hair, and a long philtrum. Neurological examination found no alterations, and physical examination yielded no relevant findings. Regarding signs of insulin resistance related to obesity, the patient had a history of hypertension, dyslipidemia, difficult glycemic control despite multimanagement, and obesity. Table 1 presents the patient’s laboratory findings from 2015 to 2022.

**Table 1:** Patient’s laboratory findings from 2015 to 2022

Year	Glycemia	Lipid profile	Renal panel test	Liver function	Hemogram	Electrolytes
2022	Fasting plasma glucose 209 mg/dL	n.d	Cr 0.8 mg/dL	n.d	n.d	n.d
2021	HBA1C 6.5%	HDL-C 53 mg/dL, TG 198 mg/dL, TC 147 mg/dL	n.d	ALT 16, AST 23	HB 14 g/dL, HTO 83%, PLT 361.000 × 10^3^/µL	n.d
	Fasting plasma glucose 267 mg/dL					
2020	HBA1C 6.47% Fasting plasma glucose 187 mg/dL	TC 233 mg/dL, HDL 52 mg/dL, TG 297 mg/dL	Microalbuminuria 1.94, RAC 11.83, Cr 0.8, BUN 27.3	n.d	n.d	Na 136 mmol/L, K 4.5 mmol/L
2019	HBA1C 6.8% Fasting plasma glucose 94	TC 218 mg/dL, TG 217 mg/dL, LDL-C 118 mg/dL	Cr 0.8 mg/dL	n.d	n.d	Na 140 mmol/L, K 4.77 mmol/L, Cl 102 mg/dL, Mg 1.92 mmol/L, Ca 10.5, P 3.2 mg/dL
2018	HBA1C 7.6%	TC 209 mg/dL, HDL 50 mg/dL, TG 480 mg/dL, LDL 118 mg/dL	BUN 24, Cr 0.81 mg/dL	ALT 65.7, AST 42.4	WBC 14,700, NEU 11.200 × 10^3^/µL, LYMPH 2320, PLT 341.0000 × 10^3^/µL	Mg 1,76 mmol/L, Ca 10,72 mg/dL, P 3.51 mg/dL
	Fasting plasma glucose 97.3 mg/dL					
2017	HBA1C 8.3% Fasting plasma glucose 168 mg/dL	TC 189 mg/dL, HDL-C 54 mg/dL, TG 277 mg/dL	Cr 0.73 mg/dL	ALT 90, AST 28	WBC 9310, HB 12.8 g/dL, HTO 41%, PLT 248.000 × 10^3^/µL	Na 137 mmol/L, K 5.09 mmol/L, Ca 10.5 mg/dL
2016	Fasting plasma glucose 242 mg/dL	TC 204 mg/dL, TG 300 mg/dL, LDL-C 102 mg/dL	Cr 0.9 mg/dL	n.d	n.d	n.d
2015	HBA1C 8.0%	TC 267 mg/dL, LDL-C 167 mg/dL, HDL-C 59 mg/dL, TG 200 mg/dL	Cr 0.83 mg/dL	n.d	n.d	n.d

*n.d.* no data, *TC* total cholesterol, *TG* triglycerides, *ALT* alanine aminotransferase, *AST* aspartate aminotransferase, *Cr* creatinine

The patient underwent an extra-institutional endocrinology follow-up since her diagnosis. When she turned 30 years old, she was referred to our clinic with obesity (BMI of 30.1 kg/m^2^) and poor glycemic control. Her initial average glycosylated hemoglobin (HbA1c) value was 8.0%, with high variability requiring Degludec insulin plus Aspart insulin up to 1 UI/kg. Despite strict follow-up and diabetes education that included bolus delivery according to carbohydrate count, only partial improvement was achieved. Her C-peptide level was measured, and its value was 0.44 ng/mL (0.5–2.0 ng/mL). Dulaglutide and dapagliflozin/metformin were initiated, achieving an HbA1c of 6.47%.

**Fig. 2:**
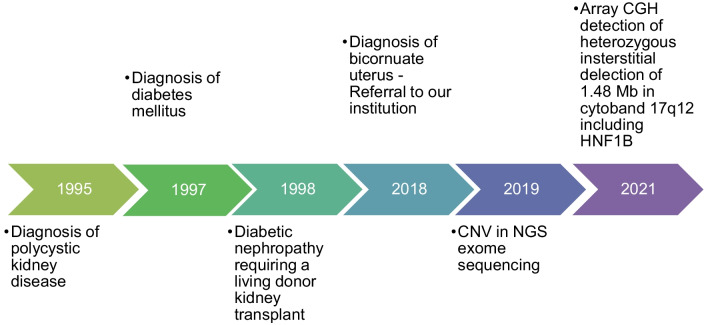
Timeline of patient’s diagnosis

Due to the phenotype of her diabetes evolution, next-generation sequencing (NGS) was performed. Exome sequencing identified copy number variants in the genes *PIGW, DDX52, AATF, C17orf78*, *MRM1, MYO19*, *TADA2A, DUSP14*,* LHX1, HNF1B,* *GGNBP2, DHRS11, ZNHIT3*, *ACACA*, and *SYNRG* associated with chromosome 17q12 deletion syndrome. This was confirmed by Array CytoScan 750 K, with detection of a heterozygous interstitial deletion of 1.48 Mb in cytoband 17q12 with genomic coordinates chr17:34,822,466–36,311,009, associated with 17q12 deletion syndrome (#614527 OMIM) [6, 7], which contributes to MODY type 5 (Fig. 2).

## Discussion

The 1.4 Mb deletion located on the long arm of chromosome 17, involving the genes *AATF, ACACA, C17orf78, DDX52, DHRS11, DUSP14, GGNBP2, HNF1B, LHX1, MRM1, MYO19, PIGW, SYNRG, TADA2A*, and *ZNHIT3* [6], explains the broad phenotype of this pathology. Patients may present with characteristic dysmorphia, such as macrocephaly, prominent forehead, full cheeks, arched brow ridges, bilateral epicanthal folds, and low nasal bridge. The deletion is also associated with MODY type 5, structural malformations in the pancreas and kidney, cholestatic liver disease, genitourinary tract malformations, cognitive deficits, and psychiatric disorders [7]. This type of deletion is usually due to alterations during the homologous recombination process, either due to balanced translocations or inversions in parents. It is associated with de novo mutations in approximately 70% of cases. It has a risk of recurrence of 50% in the patient's offspring [7, 8]. In this case, it is noteworthy that the mother, grandmother, and great-grandmother had diabetes at an early age. It could be intuited that they were carriers of a translocation that has been inherited for three generations. However, molecular studies cannot be performed to confirm the finding due to the death of relatives.

*HNF1B* belongs to the hepatocyte nuclear factor family, which is expressed in the pancreas, kidney, liver, bile ducts, and urogenital tract. It is located on chromosome 17q12 and encodes a protein that contains a homeodomain [9]. The latter is associated with a specific POU domain that acts as a transcriptional activator. *HNF1B* is a key member of the network of transcription factors controlling the differentiation of acinar, ductal, and endocrine cells [10]. It also has a dimerization domain located in the first four exons of the gene that allows it to form heterodimers with *HNF1A*. Most point mutations are described in this domain, especially in exon 2. Non-sense mutations, deletions, or insertions in this gene have been related to MODY type 5. They are associated with loss of protein function, haploinsufficiency, or negative dominance mechanisms [11, 12].

Within the broad clinical spectrum described by patients with *HNF1B* mutations, age at diabetes diagnosis was found to be greater compared to patients with T1DM (median 13.5 versus 8.8 years; *P* = 0.00001; n.s versus T2DM) [13]. In our case, the patient was diagnosed at age 11 years with rapid progression to diabetic nephropathy. It should be noted that she had a history of polycystic kidney disease diagnosed at age 9. Patients with HNF1B-MODY may present renal cysts in 62–83% of cases, pancreatic atrophy, diabetes in 48% of cases, urogenital malformations such as Mayer–Rokitansky–Küster syndrome and hypospadias, hypomagnesemia in 48% of cases, hyperuricemia, and hyperparathyroidism [12, 14] Approximately 50% of cases can be associated with deletions in this gene [15]. Therefore, in cases of suspected diagnosis, it is important to perform molecular tests such as comparative genomic hybridization to detect this type of genetic alteration.

Regarding treatment, the percentage of patients with HNF1B-MODY treated with insulin was significantly higher than that of patients with HNF4A-MODY when compared with patients with T1DM (median 65.7% versus 36.4%, *P* = 0.00001 each). This suggests that pathogenic mutations of *HNF1B* cause an earlier or more severe impairment of insulin secretion than mutations in the HNF4A gene [13]. This is consistent with the study by Brackenridge *et al.*, who demonstrated that patients with HNF1B-MODY have reduced insulin sensitivity of endogenous glucose production but normal peripheral insulin sensitivity [16]. Likewise, in the study by Horikawa *et al*., 83.3% of patients with HNF1B-MODY underwent insulin treatment from the onset of diabetes and could not be withdrawn from insulin treatment, which suggests that insulin secretion deficiency is a characteristic feature of this mutation [17]. In our case, the C-peptide level was low, which indicated impaired glucose tolerance. Warncke *et al*. compared C-peptide levels in patients with HNF1B-MODY and T1DM and showed a trend toward a higher mean level at diagnosis in the first group. However, this trend did not reach statistical significance [13]. Our patient improved her glycemic control by introducing metformin, sodium-glucose cotransporter 2 (SGLT2) inhibitors, and glucagon-like peptide-1 (GLP-1) agonists. This is concordant with studies in patients with beta cell failure (T1DM) where the efficacy of SGLT2 inhibitors and GLP-1 agonists was evaluated [18, 19].

Almost 276 cases of chromosome 17q12 deletion syndrome were reported in the literature as of 2020 [6]. In Colombia, Perdomo *et al*. described a 26-year-old male with hydronephrosis, non-insulin-dependent diabetes, and detectable C-peptide with a pathogenic 1.39 Mb deletion of 17q12 containing 20 genes, including *HNF1B*, which is different from our case with a deletion of 1.48 Mb involving 15 genes, with detectable C-peptide being an insulin-dependent diabetes patient report [20]. Thus, 17q12 deletion syndrome has different phenotypic expressions based on the genes involved, but further studies are required for clarification.

## Conclusion

The 17q12 deletion syndrome is caused by a deletion on the long arm of chromosome 17. Patients may present with diabetes and dysmorphia, such as macrocephaly, prominent forehead, full cheeks, arched brow ridges, bilateral epicanthal folds, and low nasal bridge. It is associated with diabetes in approximately 70% of cases and exhibits variable clinical presentation.

## Data Availability

The data used to support the findings of this study are restricted by the Fundación Valle del Lili Ethics Committee to protect patient privacy. Data are available from Dr. Guillermo E. Guzmán for researchers who meet the criteria for access to confidential data. The datasets used and/or analyzed during the current study are available from the corresponding author on reasonable request.
